# ABCG2, a novel antigen to sort luminal progenitors of *BRCA1*^-^ breast cancer cells

**DOI:** 10.1186/1476-4598-13-213

**Published:** 2014-09-12

**Authors:** Felicia Leccia, Luigi Del Vecchio, Elisabetta Mariotti, Rosa Di Noto, Anne-Pierre Morel, Alain Puisieux, Francesco Salvatore, Stéphane Ansieau

**Affiliations:** CEINGE-Biotecnologie Avanzate, via Gaetano Salvatore 486, 80145 Naples, Italy; Inserm UMR-S1052, Centre de Recherche en Cancérologie de Lyon, Lyon, F-69008 France; CNRS UMR5286, Centre de Recherche en Cancérologie de Lyon, Lyon, F-69008 France; Centre Léon Bérard, Lyon, F-69008 France; UNIV UMR1052, Lyon, F-69008 France; Université de Lyon, Lyon, F-69000 France; Dipartimento di Medicina Molecolare e Biotecnologie Mediche, Università di Napoli Federico II, Naples, Italy; Institut Universitaire de France, Paris, F-75000 France; IRCCS-Fondazione SDN, Naples, Italy; Laboratoire Génétique, Reproduction et Développement (GReD), CNRS UMR 6293 - INSERM U1103, Université Blaise Pascale, 24 avenenue des Landais BP80026 63171, Clermont-Ferrand, France

**Keywords:** Basal-like breast cancer (BLBC), Tumor-initiating cells (TICs), CD338/ABGG2, Antigenic phenotype

## Abstract

**Introduction:**

Tumor-initiating cells (TICs), aka “cancer stem cells”, are believed to fuel tumors and to sustain therapy resistance and systemic metastasis. Breast cancer is the first human carcinoma in which a subpopulation of cells displaying a specific CD44^+^/CD24^-/low^/ESA^+^ antigenic phenotype was found to have TIC properties. However, CD44^+^/CD24^-/low^/ESA^+^ is not a universal marker phenotype of TICs in all breast cancer subtypes. The aim of this study was to identify novel antigens with which to isolate the TIC population of the basal-A/basal-like breast cancer cell lines.

**Methods:**

We used polychromatic flow-cytometry to characterize the cell surface of several breast cancer cell lines that may represent different tumor molecular subtypes. We next used fluorescence-activated cell sorting to isolate the cell subpopulations of interest from the cell lines. Finally, we explored the stem-like and tumorigenic properties of the sorted cell subpopulations using complementary *in vitro* and *in vivo* approaches: mammosphere formation assays, soft-agar colony assays, and tumorigenic assays in NOD/SCID mice.

**Results:**

The CD44^+^/CD24^+^ subpopulation of the *BRCA1*-mutated basal-A/basal-like cell line HCC1937 is enriched in several stemness markers, including the ABCG2 transporter (i.e., the CD338 antigen). Consistently, CD338-expressing cells were also enriched in CD24 expression, suggesting that coexpression of these two antigenic markers may segregate TICs in this cell line. In support of ABCG2 expression in TICs, culturing of HCC1937 cells in ultra-low adherent conditions to enrich them in precursor/stem-cells resulted in an increase in CD338-expressing cells. Furthermore, CD338-expressing cells, unlike their CD338-negative counterparts, displayed stemness and transformation potential, as assessed in mammosphere and colony formation assays. Lastly, CD338-expressing cells cultured in ultra-low adherent conditions maintained the expression of CD326/EpCAM and CD49f/α6-integrin, which is a combination of antigens previously assigned to luminal progenitors.

**Conclusion:**

Collectively, our data suggest that CD338 expression is specific to the tumor-initiating luminal progenitor subpopulation of *BRCA1*-mutated cells and is a novel antigen with which to sort this subpopulation.

**Electronic supplementary material:**

The online version of this article (doi:10.1186/1476-4598-13-213) contains supplementary material, which is available to authorized users.

## Introduction

Breast cancer is a very heterogeneous disease, with a high degree of diversity between and within tumors. The intertumoral heterogeneity is exemplified by the identification of five molecular subtypes, namely HER2+, normal-like, luminal (subtypes A and B), basal A/basal-like and basal B/claudin-low – a classification based on gene expression profile analysis [1–5]. This heterogeneity stems from the fact that the tumor phenotype varies based on the cell of origin [6]. Indeed, basal A/basal-like and basal B/claudin-low breast cancer subtypes were reported to result from the transformation of luminal progenitors and basal/myoepithelial cells, respectively [5, 7–10]. This hypothesis has recently been challenged by the finding that a combination of several genetic events in luminal-committed cells leads to the development of breast cancers of the claudin-low subtype in murine models [11, 12]. These genetic events promote an embryonic transdifferentiation program, namely, the epithelial-mesenchymal transition (EMT), a reversible mechanism sensitive to microenvironmental changes [13]. Therefore, genetic events and the microenvironment probably constitute additional determinants of tumor etiology [14]. Intratumoral heterogeneity results from the selection of genetically distinct cell populations during tumor progression, the exacerbated plasticity of cancer cells with consequent phenotypic modifications induced by changes in the microenvironment, and the differentiation rate of the progeny of tumor-initiating cells (TICs).

Many attempts have been made to identify and characterize TICs because these cells are believed to constitute a unique sub-population with unlimited self-renewing potential that constantly fuels the tumor and sustains therapy resistance and systemic metastasis. Clarke and colleagues isolated TICs from metastatic human breast cancers based on their specific CD44^+^/CD24^-/low^/ESA^+^ antigenic phenotype [15]. However, CD44^+^/CD24^-/low^/ESA^+^ does not constitute a universal antigenic phenotype of TICs in all breast cancer subtypes [16–18]. Rather, it marks a heterogeneous mix of cells in normal mammary gland [19] and is a profile associated with cell commitment to an EMT program [20]. It is necessary to better define this antigenic phenotype by combining CD44 and CD24 with additional as yet unidentified markers or activity, as previously shown with aldehyde dehydrogenase [21]. In this context, the similar distribution of the gene expression profiles of breast cancer cell lines and primary tumors in the five subtypes identified [2, 22] suggests that the cell line diversity reflects the tumor heterogeneity. Consequently, breast cancer cell lines are considered tools with which to identify and characterize TICs. The aim of this study was to identify novel antigens that are able to isolate the TIC population of basal-A/basal-like breast cancer cell lines.

Both normal and cancer stem cells express transmembrane transporters, including ABCG2. This protein excludes the fluorescent Hoechst 33342 dye from the cells and as such, behaves as one of the major mediators of side population (SP). The SP technique has long been used to isolate both normal and cancer stem cells from different organs and species [23–28]. ABCG2 expression was found to be higher in SP cells isolated from mammoplasties of healthy patients than in non-SP cells [29]. Moreover, a specific ABCG2 inhibitor (Ko143) reduced SP formation, suggesting that ABCG2 confers the SP phenotype in mammary epithelial cells. Interestingly, SP cells, unlike their non-SP counterparts, express neither luminal nor myoepithelial markers [30], suggesting that they are dedifferentiated. In line with this observation, we demonstrate that CD338/ABCG2 is a reliable antigen with which to sort out the tumor-initiating luminal progenitor population of BRCA1-mutated breast cancer cells.

## Results

### CD338 is differentially expressed in CD24^+^and CD24^-/low^subpopulations in the *BRCA1-*mutated HCC1937 cell line

**Figure 1:**
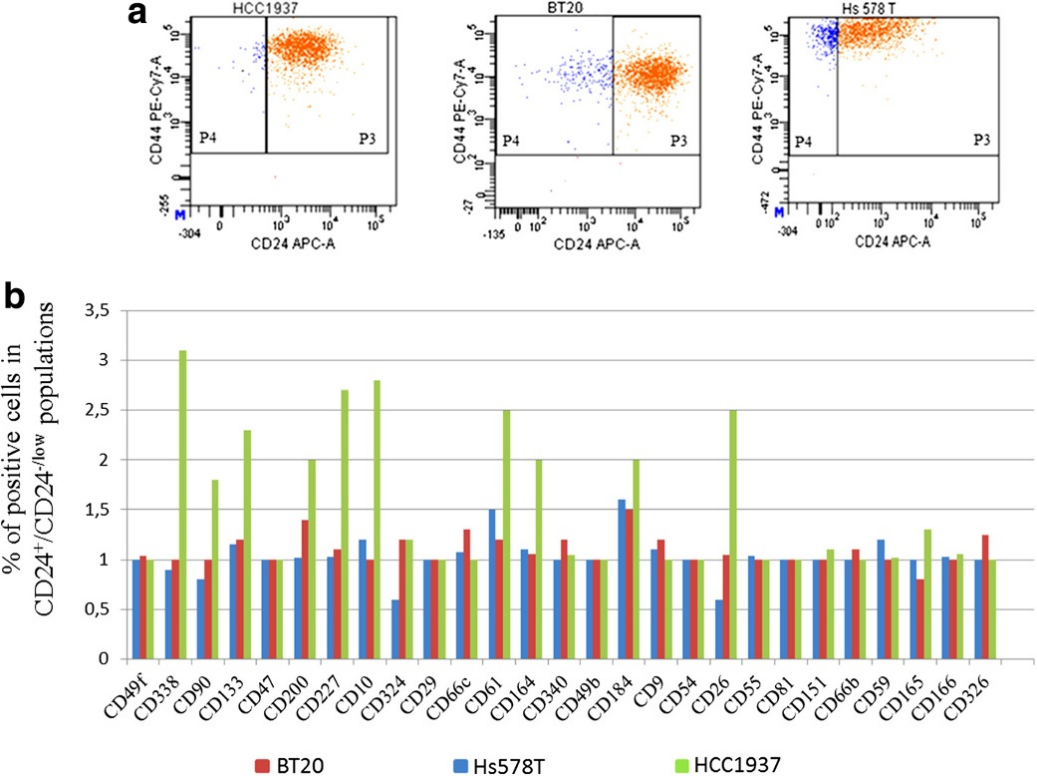
**Surface expression profile of the CD24** ^**+**^ **and CD24** ^**-/low**^ **cell subpopulations of basal-like cell lines. (a)** Expression of CD24 and CD44 in the basal A/basal-like HCC1937 and BT-20 cell lines, and in the basal B/claudin-low Hs578T cell line. CD44^+^/CD24^+^ and CD44^+^/CD24^-/low^ cell subpopulations of each cell line were defined as shown by the two gated regions, namely, P4: CD44^+^/CD24^-/low^ (blue events) and P3: CD44^+^/CD24^+^ (orange events). **(b)** The expression of 28 surface antigens was analyzed in the CD44^+^/CD24^+^ and CD44^+^/CD24^-/low^ cell subpopulations. The histogram shows the ratio between the percentage of cells positive for each antigen in the CD24^+^ and CD24^-/low^ cell subpopulations of each cell line.

To identify novel antigens that can improve the power of the CD44/CD24 antigenic phenotype in order to isolate TICs, we measured the expression of 28 surface antigens reported to be essential for cell adhesion, migration, apoptosis, cell signaling or stemness (Table 1 and Additional file 1: Table S1) in two basal A/basal-like cell lines, namely BT20 and HCC1937 (*BRCA1*^-/-^) and the basal B/claudin-low Hs578T cell line. Figure 1 shows the expression of these antigens, as assessed by flow-cytometry, in the CD44^+^/CD24^+^ and CD44^+^/CD24^-/low^ cell subpopulations. Particularly, we determined the ratio between the percentage of cells positive for each antigen in the two cell subpopulations of each cell line (Figure 1b). No significant differences were observed in the expression of the examined antigens between the CD44^+^/CD24^+^ and CD44^+^/CD24^-/low^ cell subpopulations in the BT20 and Hs578T cell lines (ratio ± 1), while several of them were significantly enriched in the CD44^+^/CD24^+^ population of HCC1937, including the stemness markers CD10, CD133 and CD338/ABCG2 [25, 31, 32]. Evaluation of the mean fluorescence intensity (MFI) of each surface marker in the CD44^+^/CD24^+^ and CD44^+^/CD24^-/low^ cell subpopulations of the HCC1937 cell line, demonstrated that CD338 is expressed at a higher level in the CD24^+^ than in CD24^-^ cell subpopulation (Additional file 2: Figure S1).

**Table 1:** **Molecular identity and functions of the antigens analyzed by flow-cytometry**

CD	Molecule	Function	References
**CD9**	P24	Cell adhesion and migration	[42]
**CD10**	CALLA	Antigen overexpressed in many tumors	[43]
**CD24**	HSA	Adhesion and metastatic tumors	[15]
**CD26**	DPPIV	Exopeptidase, tissue restructuring	[44]
**CD29**	β1 integrin	Adhesion to matrix proteins	[29]
**CD44**	H-CAM	Cell polarity, suppression of apoptosis, metastasis	[15]
**CD47**	Rh-associated protein	Cell activation, apoptosis, cell spreading	[45]
**CD49b**	α2 integrin	Cell adhesion to collagen and laminin	[46]
**CD49f**	α6 integrin	Cell adhesion, migration, cell surface signaling	[47]
**CD54**	ICAM	Cell adhesion, immune reactions	[48]
**CD55**	DAF	Protection against complement	[49]
**CD59**	MIRL	Protection from complement-mediated lysis	[50]
**CD61**	β3 integrin	Cell adhesion, cell signaling	[29]
**CD66b**	CEACAM8	Cell adhesion, cell signaling	[51]
**CD66c**	CEACAM6	Cell adhesion, cell signaling	[51]
**CD81**	TAPA-1	Response to antigens	[52]
**CD90**	Thy-1	Cell adhesion and differentiation	[53]
**CD105**	ENG (Endoglin)	Angiogenesis, vessel wall integrity	[54]
**CD133**	Prominin 1	Unknown	[32]
**CD151**	PETA-3	Cell adhesion	[55]
**CD164**	MGC-24	Adhesion and homing	[56]
**CD165**	AD2	Unknown	[57]
**CD166**	ALCAM	Adhesion, organ development	[58]
**CD184**	CXCR4	Increased expression in mammospheres	[59]
**CD200**	OX2	Immunosuppression	[60]
**CD227**	MUC1	Response to hormones and cytokines	[31]
**CD324**	E-Cadherin	Cell adhesion, tumor suppression	[61]
**CD326**	EpCAM	Cell adhesion	[31]
**CD338**	ABCG2	Pumping cytotoxic drugs out of cells	[62]
**CD340**	Her2/neu	Cell growth and differentiation	[63]

**Figure 2:**
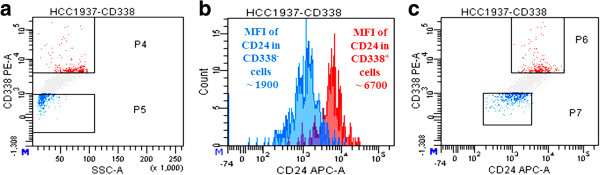
**Co-segregation of a unique subcellular population with CD338 and CD24.** The mean fluorescence intensity of CD24 was 4.7-fold higher in CD338^high^ cells (panel a, red events) than in CD338^-^ cells (panel **a**, blue events; mean ± SEM: 5,200.0 ± 916.5 and 1,100.0 ± 404.1, p < 0.05), as shown by a monoparametric histogram of CD24 expression (panel **b**) and by CD338 *vs* CD24 dot plot (panel **c**).

Given the high differential of CD338 (ratio >3, Figure 1), we explored the link between CD24 and CD338 expression. To this aim, we gated the HCC1937 CD338^high^ and CD338^-^ cell subpopulations, and measured CD24 expression. As shown in Figure 2, the MFI was 7-fold higher in CD338^high^ cells (panel a, red events) than in CD338^-^ cells (panel a, blue events; mean ± SEM: 5,200.0 ± 916.5 and 1,100.0 ± 404.1, p < 0.05). This finding is consistent with a overlap between CD24^+^ and CD338^high^ cells.

### Enrichment in stem cells parallels an increase in CD338 expression

**Figure 3:**
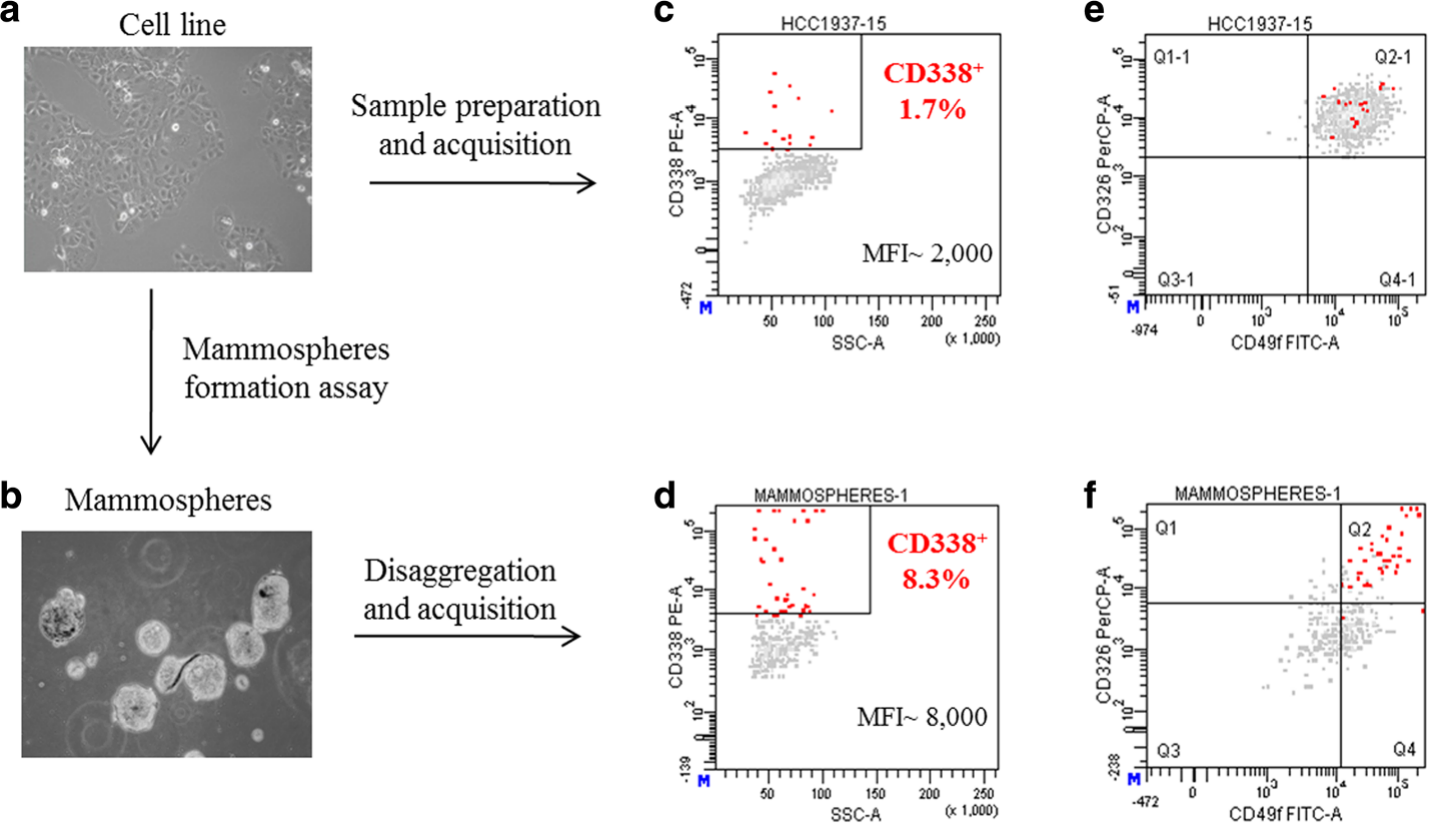
**Culture in ultra-low adherent conditions enriches in CD338-expressing cells.** HCC1937 cells were either cultured in adherent (panel **a**) or ultra-low adherent conditions (panel **b**). CD338 expression was assessed on adherent cells (panel **c**) and in third-generation mammospheres (panel **d**). CD326 and CD49f expression in the adherent cell line (panel **e**) and on mammosphere-derived cells (panel **f**) was assessed.

If CD338 is a reliable marker of TICs, culture conditions reported to induce enrichment of stem cells and progenitors should be associated with an increase in CD338 expression. To address this issue, we performed mammosphere formation assays in which we ran three successive culture/dissociation passages in ultra-low adherent conditions, and measured the percentage of CD338-expressing cells. The percentage of CD338^high^ cells in mammospheres was 4.9-fold higher than that in the whole cell line when cultured in adherent conditions (mean ± SEM: 8.3% ± 0.5 and 1.7% ± 0.1 respectively, p < 0.0001). Furthermore, the MFI of CD338 cells, which indicates its expression level, was 3.8-fold higher in mammosphere-derived cells than in adherent cells. This observation supports the assumption that CD338-positive cells display some stem cell-like properties (Figure 3). Strikingly, CD338-positive cells in ultra-low adherent conditions were the only cell subpopulation to retain CD326/EpCAM and CD49f/α6-integrin expression (Figure 3f), which is an antigenic phenotype assigned to luminal progenitors.

### CD338-expressing cells display stemness properties and transformation potential

**Figure 4:**
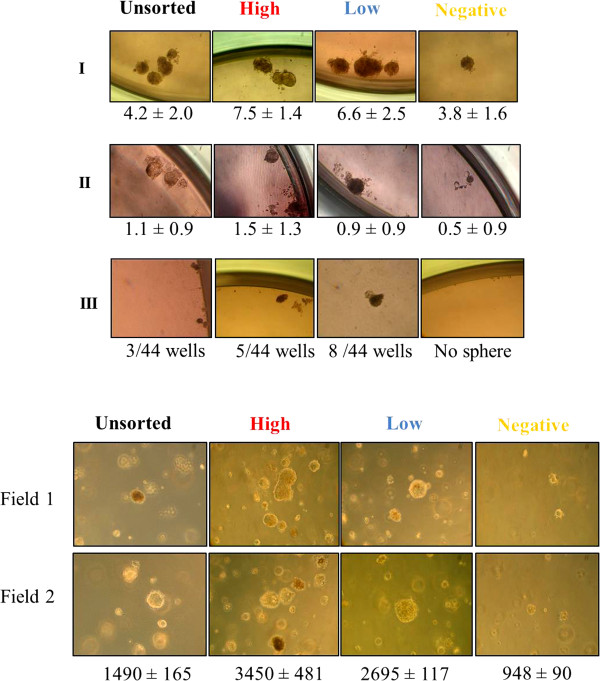
**CD338 expression discriminates cells with stemness properties and transformation potential. (a)** Mammosphere formation assay. CD338^high^, CD338^low^ and CD338^-^ sorted sub-populations of HCC1937 cells were plated in ultra-low adherent conditions at a low density to generate mammospheres. Upper (I), central (II) and lower (III) panels show the results of the first-, second- and third-generation mammospheres, respectively. Results are expressed as mammosphere-forming efficiency (MFE, number of mammospheres/number of wells) ± SD of triplicates. **(b)** Soft agar colony formation assay. CD338^high^, CD338^low^, and CD338^-^ cells were sorted out from HCC1937 cells and tested for their ability to generate colonies on soft-agar. The number of colonies observed after 4 weeks are indicated for 5 × 10^4^ plated cells ± SD of triplicates.

If CD338 is an antigenic marker of TICs in the HCC1937 cell line, the CD338^high^ cellular subpopulation would be expected to generate mammospheres when cultured in ultra-low adherent conditions, whereas CD338^-^ cells should be devoid of stemness properties. To test this hypothesis, we sorted by flow-cytometry (Additional file 3: Figure S2) three distinct populations that differ in the expression of CD338: a CD338^high^ population expressing CD338 at high level (1% of cells), a CD338^low^ population expressing CD338 at low level (79% of cells), and a CD338^-^ population negative for CD338 (20% of cells). The three sorted populations were grown for two days in standard adherent conditions to allow them to recover from the sorting procedure before testing their stemness properties in a mammosphere formation assay. As expected, after two serial passages, only CD338-expressing cells gave rise to mammospheres (Figure 4a).

**Figure 5:**
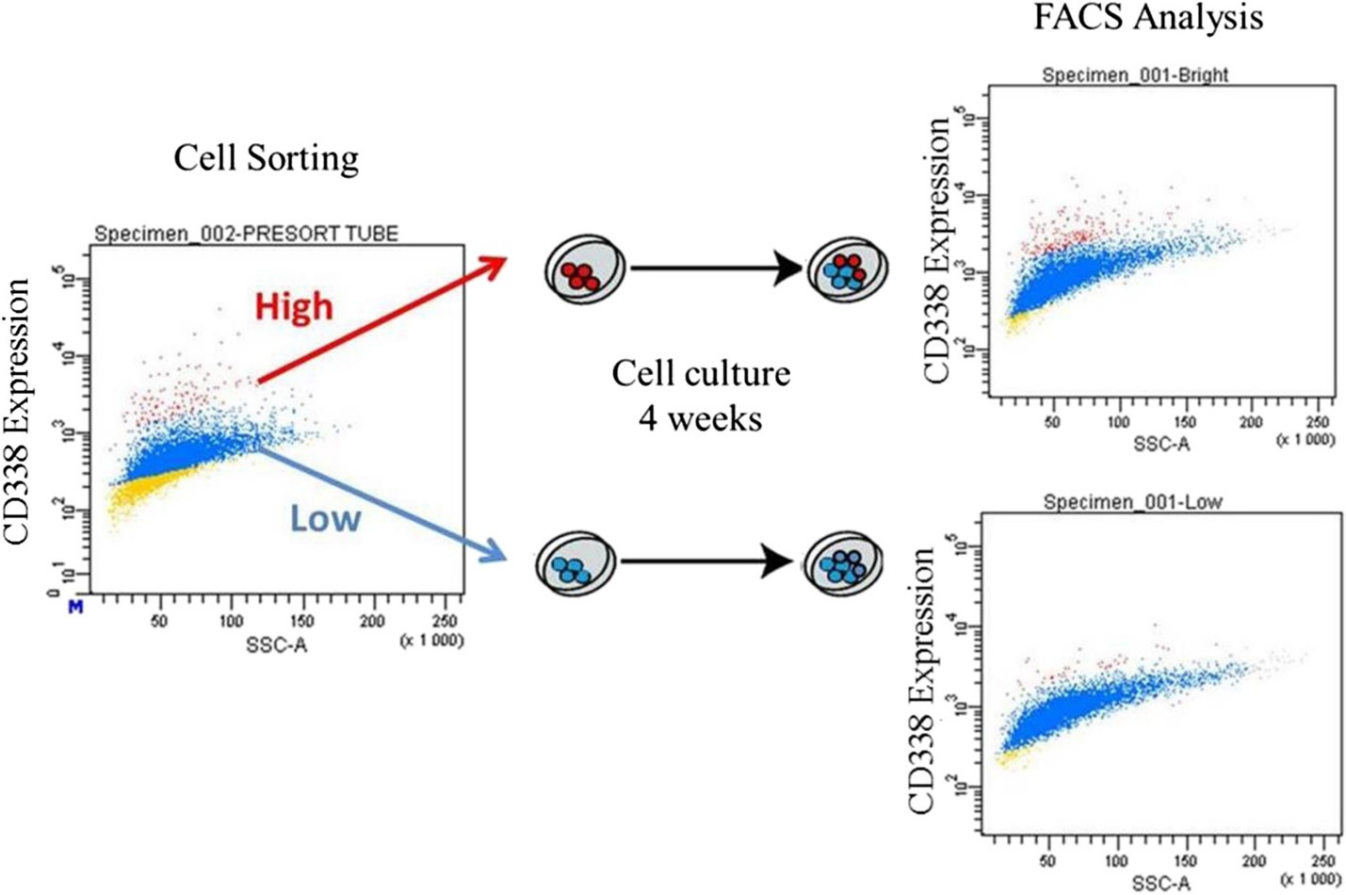
***In vitro*** **evolution of CD338** ^**high**^ **and CD338** ^**low**^ **sorted populations.** CD338^high^ and CD338^low^ cells were sorted out from HCC1937 cells and plated in adherent conditions. After four weeks of culture, the expression of CD338 was analyzed by flow-cytometry.

We next assessed the transformation potential of the three subpopulations in a soft-agar colony assay. As shown in Figure 4b, CD338-positive cells, CD338^high^ and CD338^low^, displayed a significantly higher transformation potential than CD338^-^ cells. The few colonies observed in CD338^-^ cells probably reflect contamination of the cell population with CD338^low^ cells during the cell sorting (Additional file 4: Figure S3). There were no significant differences between CD338^high^ and CD338^low^ populations in either the mammosphere or the and colony formation assay. Assays were invariably performed shortly after reseeding sorted cells. Notably, after several days in culture, CD338^high^ cells gave rise to a heterogenous CD338^high^ and CD338^low^ population, which suggests parenting between these cells (Figure 5). Both CD338^high^ and CD338^low^ cells, but not CD338^-^ cells, displayed stemness properties and transformation potential.

**Figure 6:**
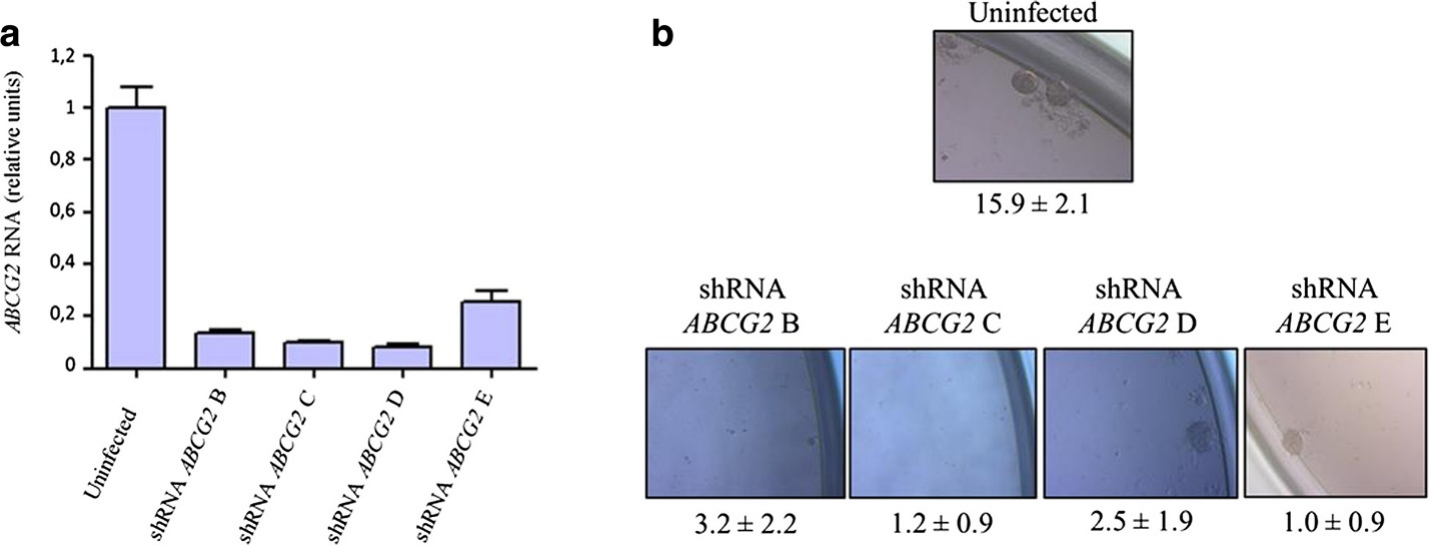
**The knockdown of ABCG2 annihilates the stemness properties of HCC1937 breast cancer cells.** ABCG2 expression was turned down through RNA interference and the stemness properties of the resulting cell lines was examined. **(a)** Assessement of ABCG2 expression by q-RT-PCR. Levels expressed relatively to the housekeeping HPRT1 gene transcripts were normalized with respect to uninfected HCC1937 cells. **(b)** Mammospheres formation assay. Results of second generation mammospheres are shown and expressed as mammosphere-forming efficiency (MFE, number of mammospheres/number of wells) ± SD.

To strengthen our conclusions, we next assessed the consequences of ABCG2 depletion on stemness properties of HCC1937 breast cancer cells by performing mammospheres formation assays. In line with our expectations, the knockdown of *ABCG2*, achieved through RNA interference, leads to a significant decrease of stemness potentials (Figure 6).

### CD338^high^cells display a selective advantage *in vivo*

**Figure 7:**
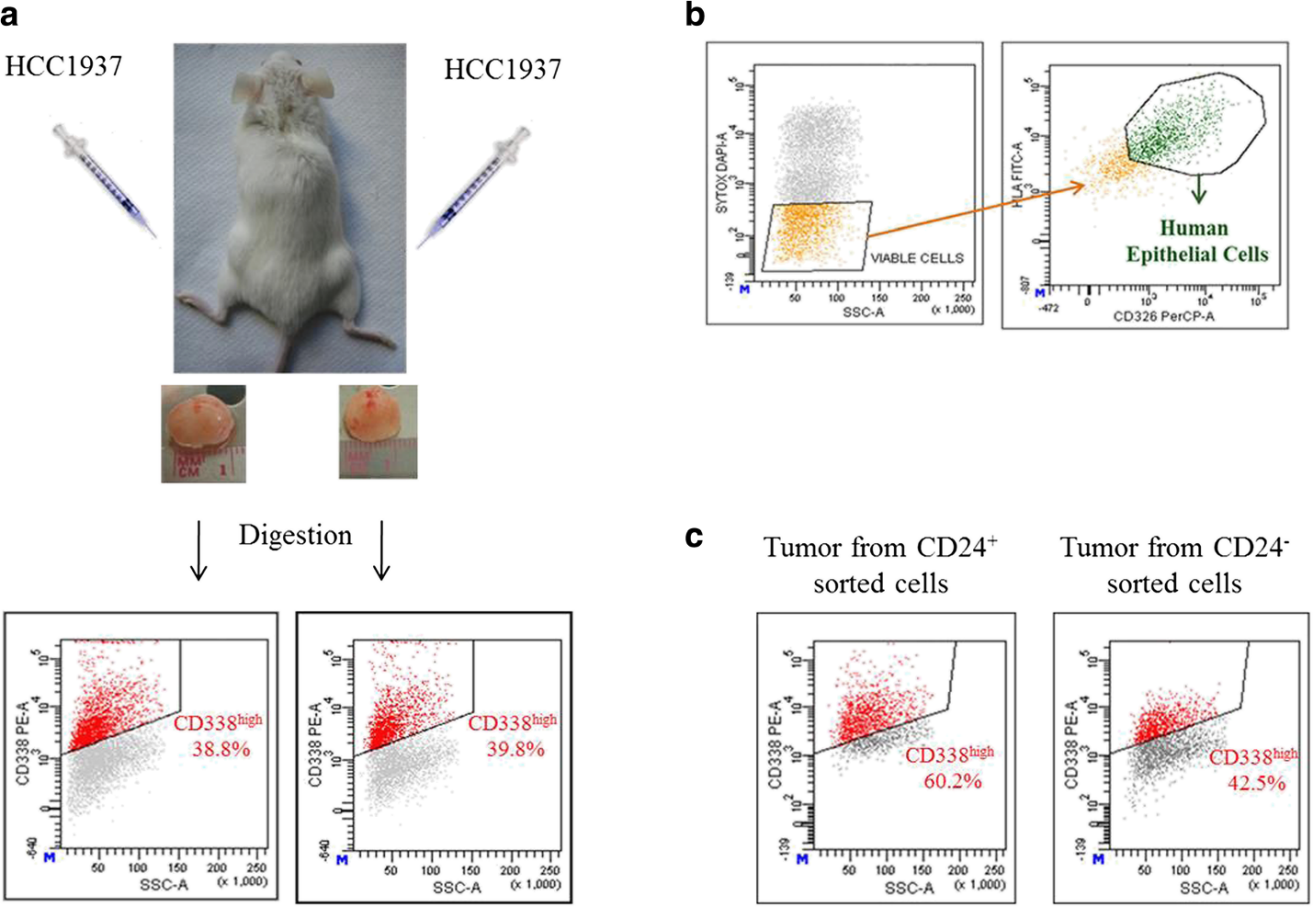
**CD338-positive cells display a selective advantage** ***in vivo*** **. (a)** HCC1937 cells were injected subcutaneously into the left and right flanks of five NOD/SCID mice. Dot plots show the percentages of CD338^high^ cells in the cell suspensions obtained from digestion of tumor tissues. **(b)** Gating strategy to analyze by flow-cytometry the expression of CD338 cells in the excised tumors. Percentages of the CD338^high^ cell subpopulation were determined in viable (SYTOX^-^), human (HLA-ABC^+^), epithelial (EpCAM^+^) gated cells. **(c)** Enrichment of CD338-positive cells in CD24^+^-cell derived tumors. HCC1937 CD24^+^ and CD24^-^ cell subpopulations were xenografted into NOD/SCID mice. CD338^high^ expressions were assessed in the viable (SYTOX^-^) human, (HLA-ABC^+^), epithelial (EpCAM^+^) gated tumor-derived cells.

Because the tumorigenic potential is specific to TICs, we assumed that, when xenografted into immunocompromised mice, HCC1937 cell-generated tumors would be enriched in CD338-expressing cells. To address this point, 2 × 10^6^ and 4 × 10^5^ HCC1937 cells were injected into the left and right flanks of NOD/SCID mice, respectively (Figure 7a). Invariably, cells induced tumor formation with a delay depending on the number of cells injected (100%, n = 5). Tumors were excised, digested to single cell suspensions and analyzed by flow-cytometry. The percentages of CD338^high^ cells were determined in viable (SYTOX^-^), human (HLA-ABC^+^), epithelial (EpCAM^+^)-gated tumor-derived cells (Figure 7b). The CD338^high^ subpopulation was significantly enriched in both tumors (38.8% ± 1.1) compared with the parental cell line (Figure 7a). To compare the tumorigenic potential of the CD24^+^ and CD24^-^ cell subpopulations, we sorted the two populations and immediately xenografted them into mice. The same number of cells (assays were performed with either 5 × 10^4^ or 5 × 10^5^ cells) were injected into the flanks of five mice. All injections invariably led to tumor growth and there were no obvious differences between the CD24^+^ and CD24^-^ cell subpopulations. Moreover, no difference in tumor growth was detected between CD24 sorted cells and the unsorted HCC1937 cell line (data not shown). While the CD24^+^ and CD24^-^ cell populations displayed a similar tumorigenic potential, CD24^+^-derived tumors had a higher percentage of CD338^high^ cells than CD24^-^-derived tumors (60.2% ± 3.4 versus 42.5% ± 0.5) (Figure 7c). This observation strengthens the link between CD24 and CD338 expression.

## Discussion

In an attempt to identify novel antigens that may be combined with CD44 and CD24 to specifically sort TICs, we compared the expression of a panel of surface antigens between the CD44^+^/CD24^-/low^ and CD44^+^/CD24^+^ cell subpopulations of three basal A or B breast cancer cell lines. Neither the stemness-associated CD10 and CD133 antigens nor ABCG2 differed between the CD44^+^/CD24^-/low^ and CD44^+^/CD24^+^ cell subpopulations of the BT20 and Hs578T cell lines. This finding questions the reliability of CD44/CD24 in identifying TICs in these two cell lines. Conversely, in the *BRCA1*-mutated HCC1937 cell line, which was previously reported to include a TIC population that does not display a CD44^+^/CD24^-/low^ antigenic phenotype [33], the CD44^+^/CD24^+^ subpopulation displayed significantly increased expression in several stemness markers particularly the ABCG2 transporter (i.e., the CD338 antigen). The positive correlation between CD24 and CD338 is confirmed by enrichment of CD338-overexpressing cells in tumors originating from CD24-positive cells subcutaneously injected into mice (Figure 7c).

In support of ABCG2 expression in TICs, cell culture conditions that induced enrichment of stem/progenitor cells (mammosphere formation assays) were associated with a significant increase in CD338 level. Since CD338 is one of the major mediators of SP, the enrichment in CD338^+^ cells as observed in mammospheres is in line with the previously reported enrichment in SP cells in mammopsheres [34]. The latter SP was reported to contain bipotent progenitors and stemness properties as judged by their ability to generate mammospheres when cultured in ultra-low adherent conditions [34]. We thus investigated the reliability of this antigen in sorting TICs in the context of this cell line, and found that the stemness and transformation potentials were specifically assigned to the CD338-expressing cell subpopulation. Furthermore, the CD338^+^ subpopulation was significantly increased in tumors arising from HCC1937 cells subcutaneously xenografted into immunocompromised mice. The transformation potential was previously assigned to the CD24^+^ subpopulation of HCC1937 cells [33]. Given the good correlation between the expression of the CD338 and CD24 surface antigens, we conclude that the CD24^+^/CD338^+^ cells likely include HCC1937 TICs.

The enrichment of the CD338^high^ subpopulation in HCC1937-derived tumors versus the parental cell line suggests that CD338^high^ cells have an adaptative advantage *in vivo*. Notably, by studying the MDA-MD-435 basal A cell line, Patrawala and colleagues reported that, although ABCG2 preferentially marks proliferating cells, the ABCG2^+^ and ABCG2^-^ cell subpopulations display a similar tumorigenic potential [35]. This discrepancy either reflects different ABCG2 expression profiles in the two cell lines or the presence in MDA-MB-435 cells of other transporters with redundant functions.

Single markers are not sufficient to identify and isolate stem cells. This is supported by the observation that tumorigenic assays that we performed with CD24^+^ and CD24^-^ sorted cells did not reveal any significant differences in the tumorigenicity between the two CD24 sorted populations. Therefore, CD24 alone is not sufficient to specifically sort stem cells. In this context, CD338 is more stringent and the combination of the two antigens probably results in a better segregation, since the increase of CD338^high^ cells in tumor tissues originated from a CD24^+^ sorted cell population. Moreover, mammosphere formation assays revealed that, among CD24^+^ cells, those overexpressing CD338 displayed a higher mammosphere forming efficiency (Additional file 5: Figure S4). The higher specificity of CD338 than CD24 to mark TICs in the HCC1937 cell line is further supported by the fact that CD338 was enriched in xenograft tumors (Figure 7) whereas CD24 and CD44 expression profiles did not change *in vivo* (data not shown).

CD338 expression analysis revealed three distinct cellular subpopulations. While CD338^-^ cells probably include non-tumorigenic and differentiated cells, the significance of the two CD338^+^ cell populations (CD338^high^ and CD338^low^) remains elusive. It is conceivable that CD338^low^ cells arise from CD338^high^ cells, thereby forming two separate populations.

Asymmetric division is one of the main properties of stem cells [36, 37]. The analysis of CD338^high^ and CD338^low^ sorted sub-populations, after culture for several weeks, revealed that the antigenic phenotype of CD388^low^ cells remained stable and homogeneous, whereas CD338^high^ cells gave rise to CD388^high^ and CD388^low^ cells which suggests filiation of CD338^low^ cells from CD338^high^ cells (Figure 5). Our results are in agreement with the findings of Patrawala and colleagues who found that, in several tumor cell lines, 1% of the ABCG2^+^ dividing cells segregated asymmetrically [35]. This observation suggests that a small proportion of ABCG2^+^ cancer cells (likely corresponding to the CD338^high^ subpopulation in the HCC1937 cell line) might divide asymmetrically. In support of Patrawala’s observation, we demonstrate that CD338^+^ HCC1937 cells proliferate faster than their CD338^-^ counterparts (Additional file 6: Figure S5). It is thus likely that ABCG2 marks proliferating cells and some of them (very likely the CD338^high^ subpopulation) might undergo asymmetrical divisions, a feature of stem cells.

Despite some differences in *ABCG2* transcript levels (Additional file 3: Figure S2b), it is also feasible that parenting results from the dynamic expression of CD338 [38], through protein internalization.

Cell sorting experiments were performed by using the monoclonal anti-CD338 antibody 5D3. 5D3 binding to its extracellular epitope strongly depends on the conformation of ABCG2 [39], making the binding particularly unstable. The stability of the binding was further affected by the very long cell sorting, as the isolation of the rare CD338^high^ population (1% of the whole cell line) took between 7 to 9 hours. Attempts to stabilize the antibody-antigen interaction with a protein cross-linker (PMPI, p-Maleimidophenyl isocyanate) successfully increased the purity of CD338^high^ sorted cell subpopulation (from 50-70% to 90-95%; Additional file 7: Figure S6a). Unfortunately, the crosslinking of CD338 down-modulated its activity as demonstrated by the lack of colonies when unsorted cells were plated on soft agar (Additional file 7: Figure S6b).

*BRCA1*-mutated basal-like breast cancers are believed to arise from a developmental stage of the mammary epithelial cell, which is different from the primitive stem cell, named the luminal progenitors [7, 8, 40, 41]. It is noteworthy that CD338^+^ cells in mammosphere-derived HCC1937 cells are the only cell subpopulation that maintained the expression of CD326/EpCAM and CD49f/ α6-integrin, a combination of antigens previously assigned to luminal progenitors [7]. Collectively, our data suggest that CD338 is specific to the luminal progenitor subpopulation of *BRCA1*-mutated cells and is a novel antigen with which to sort this important subpopulation.

## Conclusion

Since the CD44^+^/CD24^-/low^ antigenic phenotype does not constitute a universal antigenic phenotype of TICs in all breast cancer subtypes, it is necessary to identify novel TIC markers in order to better define this phenotype. Particularly, *BRCA1*-mutated basal-like breast cancers are believed to arise from the luminal progenitors [7, 8, 27, 28]. Here, we have identified an additional reliable antigen, CD338/ABCG2, that can be used to refine the sorting of the luminal progenitor subpopulations of *BRCA1*-mutated breast cancer cells.

## Methods

### Cell lines

The human breast cancer cell lines Hs578T and BT-20 were provided by American Type Culture Collection (Rockville, MD, USA) and cultured in DMEM, 10% FBS. The HCC1937 cell line was from the American Type Culture Collection, and was cultured in IMDM medium (Invitrogen), 20% FBS (Gibco).

### Flow cytometry analysis and cell sorting

#### Antigens and antibodies

Multi-color flow-cytometry was performed with anti-human monoclonal antibodies (MoAbs) that were conjugated with phycoerythrin (PE), fluorescein isothiocyanate (FITC), phycoerythin-Cy7 (PE-Cy7) or Alexa Fluor 647. The study was performed with the following antibodies: PE-conjugated MoAbs against CD10, CD29, CD54, CD55, CD59, CD61, CD151, CD166, CD200, CD340 and FITC-conjugated MoAbs against CD9, CD26, CD47, CD49b, CD49f,CD66b, CD66c, CD81, CD164, CD165, CD227, and CD326 (BD Biosciences); FITC-conjugated antibody against CD90 and CD324 (BD Pharmigen); PE-coniugated MoAb against CD133 (Miltenyi Biotech); AlexaFluor647-conjugated MoAb against CD24, PE-Cy7-conjugated MoAb against CD44 and PE conjugated MoAb against CD338 (Biolegend); PE-conjugated MoAb against CD184 (Immunotech); PE-conjugated MoAb anti-CD105 (Serotec).

#### Flow-cytometry panel and sample preparation

We used a four-color flow-cytometry panel to measure the expression of the 28 surface markers in addition to CD44 and CD24 [15, 29, 31, 32, 42–63]. Cells were stained with anti-CD24-AlexaFluor 647 and anti-CD44-PECy7 monoclonal antibodies combined with pairs of antibodies, conjugated with two other different fluorochromes (PE and FITC), directed against the additional surface antigens examined (Additional file 1: Table S1). An analysis buffer (RPMI without red phenol (Invitrogen), 1-2% FBS (Gibco), 10 U/ml DNase (Sigma-Aldrich)) was used to prepare cells for analysis. Enzymatically individualized cells were counted, resuspended in the analysis buffer at 5 × 10^6^/ml and stained by incubation at 4°C for 20 min with the appropriate MoAbs. For FACS analysis, cells were stained in a 100 μl labelling volume and the concentrations of labelling antibodies were: 0.1:10 for CD44, 2:10 for CD338 and 0.5:10 for all other antibodies. Samples were washed twice with the analysis buffer, centrifuged and resuspended in 0.5 ml of FACS flow sheath fluid (BD Biosciences). Immediately before FACS acquisition, cells were incubated at room temperature in the dark with SytoxBlue (Invitrogen) or DAPI (Invitrogen) to exclude dead cells. All experiments included a negative control to exclude the signal background caused by the cellular auto-fluorescence.

For fluorescence-activated cell sorting, HCC1937 cells were enzymatically individualized, resuspended at 5 × 10^7^ cells/ml in sorting buffer (RPMI without red phenol (Invitrogen), 1-2% FBS (Gibco), 10 U/ml DNase (Sigma-Alderich), 2.5 mM EDTA) and stained by incubation with CD338 and/or CD24 MoAbs at 4°C for 1 h. After staining, samples were washed twice with sorting buffer, and resuspended at 2 × 10^7^ cells/ml in SB. Cells were sequentially filtered (50 μm, Partech) and incubated for a few minutes at room temperature in the dark with a vital dye, SytoxBlue (Invitrogen) or DAPI (Invitrogen).

#### Cytometers

The samples were analysed on a FACSAria I flow-cytometer (Becton Dickinson, Franklin Lakes, NJ, USA) (Figures 1, 2 and 3; Additional file 8: Figure S7). FITC, PE and PE-Cy7 fluorescence was determined by a 488 nm excitation line and detected by 530/30 nm, 585/42 nm and 780/60 nm filters, respectively. AlexaFluor-647 fluorescence was determined by a 633 nm excitation line and detected by a 660/20 nm filter. For each sample run, 10^4^ to 2 × 10^4^ events were recorded and analysed.

The expression of CD338 was also analyzed with the BD LSR II four-laser flow-cytometer (Becton Dickinson, Franklin Lakes, NJ, USA) (Additional file 3: Figure S2 and Additional file 4: Figure S3) by exciting PE fluorochrome with the 561 nm laser. This laser enabled us to discriminate three cell subpopulations based on the expression of CD338: CD338^neg^, CD338^low^ and CD338^high^.

Live cell sorting experiments were performed using BD FACSAria I with 100 μm nozzle. PE fluorescence of CD338 was determined by a 488 nm excitation line and detected by 585/42 nm filters, whereas Alexa-Fluor 647 fluorescence of CD24 was determined by a 633 nm excitation line and detected by 660/20 nm filters. Sorted cells were collected in RPMI medium without red phenol (Invitrogen), 20% FBS (Gibco) 10 U/ml DNase (Sigma-Alderich) and 2% penicillin/streptomycin (Invitrogen) collecting buffer. Cell sorting of CD338 populations took 7 to 9 hours, whereas cell sorting of the CD24 populations took 30 to 90 minutes. Sorted populations were xenografted in mice immediately after cell sorting. An example of CD24 sort purity is reported in (Additional file 9: Figure S8).

#### Analysis of cytometric data

The samples were analyzed using the FACSDiva software (Becton Dickinson). We used a three-gating strategy to define the target cell population to analyze the expression of the 28 surface markers (Additional file 8: Figure S7a, b, c). First, to exclude dead cells and debris, cells were gated on a two physical parameters dot plot measuring forward scatter (FSC) versus side scatter (SSC). Second, we excluded doublets by gating cells on FSC-height versus FSC-area dot plots. Lastly, to exclude dead cells, we gated Sytox-Blue- or DAPI-negative cells. The expression of each surface marker in the different cell lines was reported as percentage of positive cells in Count versus FITC- or PE-CD histograms (Additional file 8: Figure S7d). We also measured the expression level of each antigen in the different CD44/CD24 subpopulations, CD44^+^/CD24^-/low^ and CD44^+^/CD24^+^, since each FITC-CD or PE-CD antibody was combined with anti-CD44 and anti-CD24 antibodies (Additional file 2: Figure S1).

### ABCG2 knockdown

shRNA ABCG2 lentiviral particles were generated through co-transfection of 293 T cells with 4 different shRNA pLKO.1 (4 different shRNAs GCAACAACTATGACGAATCAT, CCTTCTTCGTTATGATGTTTA,GCTGTGGCATTAAACAGAGAA,CCTGCCAATTTCAAATGTAAT, Sigma Aldrich), pCMV R8.91 (gag-pop-Tat-Rev) and phCMVG-VSVG (env) expression constructs using the calcium phosphate precipitation technique. Infections were performed as described in [64]. Infected cells were selected with puromycin (1 μg/ml) before being plated in ultra-low adherent conditions or plated on soft agar. Inhibition of ABCG2 expression was confirmed by qRT-PCR.

### Mammosphere formation assays

For mammosphere generation, HCC1937 cells were seeded in 96-well ultra-low attachment plates (Corning, New York, NY, USA) at the concentration of 1,000 cells/well for first-generation mammospheres, and at 100 cells/well for subsequent passages. Cells were grown in a serum-free mammary epithelial growth medium (MEBM, Lonza, Verviers, Belgium) supplemented with B27 (Invitrogen, Carsbal, CA, USA), 20 ng/ml EGF, 20 ng/ml bFGF and 4 μg/ml heparin (Sigma, St. Louis, MO, USA). Mammospheres were collected by gentle centrifugation (1,000 rpm, 5 minutes), and enzymatically (5 min in trypsin/EDTA) and mechanically (26 Gauge needle) dissociated between each step and for further analysis.

### Soft agar colony formation assay

Plates were coated with 0.75% low-melting agarose (Lonza) obtained by mixing equal volumes of 1.5% agar and 2× growth medium (IMDM). Cells were enzymatically dissociated, resuspended in growth medium and counted. An overlaid suspension of cells in 0.45% low-melting agarose was obtained by mixing equal volumes of 0.9% agar and 2 × IMDM with cells (5 × 10^4^ cells/well in 6-well plates). Plates were incubated for 3–4 weeks at 37°C and colonies were counted under microscope.

### *In vivo*tumorigenicity assays

All animal experiments were conducted in accordance with accepted standards of animal care and in agreement with a protocol established with our Ethics Committee. The study has been approved by the Institutional Review Board “CEINGE-Biotecnologie Avanzate”. To evaluate *in vivo* tumorigenicity, unsorted HCC1937 or sorted cell subpopulations were resuspended in media and Matrigel (1:1; BD Biosciences), and injected into the left and right flanks of 4-week-old NOD/SCID mice (C. River laboratories). To evaluate the tumorigenic potential of the unsorted cell line, 2 × 10^6^ and 4 × 10^5^ of HCC1937 cells were injected into the left and right flanks of mice respectively (n = 5). To compare the tumorigenic potentials of the CD24^+^ and CD24^-^ cell populations, 5 × 10^4^ or 5 × 10^5^ cells were injected into the left and right flanks of NOD/SCID mice respectively (n = 5). Tumor formation was assessed and measured once a week. Animals were killed when the tumor reached the size of 1.5 cm. Tumor tissues were minced into < 1 mm pieces, dissociated in an enzymatic solution consisting of collagenase type 1 (1.5 mg/ml, Sigma), penicillin/streptomycin 20%, amphotericin 1% and DNase (1 mg/ml, Roche), and incubated at 37°C for 60 min with gentle agitation. The single cell suspensions were analyzed by flow-cytometry after staining with appropriate antibodies.

## Electronic supplementary material

Additional file 1: Table S1: Four-color flow cytometry panel for the expression analysis of surface markers in BT20, HS578T and HCC1937 breast cancer cell lines. (PDF 4 KB)

Additional file 2: Figure S1: Expression of surface antigens in the CD24^+^ and CD24^-/low^ cell subpopulations of the HCC1937 cell line. The surface expression of the breast cancer cell lines was evaluated using a four-color flow-cytometry panel. In particular, we stained cells with monoclonal antibodies against the two classical breast cancer stem cell markers, CD44 and CD24, combined with pairs of antibodies against molecules explored as potential novel TICs markers. Hence, we analyzed and compared the expression of 28 surface markers in the CD44^+^/CD24^-/low^ and CD44^+^/CD24^+^ cell subpopulations of each cell line. This figure shows the ratio of the mean fluorescence intensity (MFI) of each antigen evaluated in the two cell subpopulations, CD24^+^ and CD24^-^, present in the HCC1937 cell line. Most CDs displayed a ratio near to 1, which indicated an equal or very similar expression in the two cell subpopulations, whereas CD338/ABCG2 and CD10/CALLA, which are two known stem cell markers, displayed a ratio greater than 2, which indicated that they are expressed at a higher level in the CD24^+^ than in CD24^-^ cell subpopulation. (PDF 143 KB)

Additional file 3: Figure S2: Expression of CD338 in the HCC1937 cell line and cell sorting of three cell subsets. (a) Using the LSR II cytometer, we identified three distinct CD338 subpopulations: 1) CD338^+/high^ (red events) expressing CD338 at high level and consisting just in 1% of the total cell line; 2) CD338^neg^ (yellow events) not expressing CD338 and constituting about 20% of the total cell line; and 3) CD338^+/low^ (blue events) expressing CD338 at an intermediate level and constituting about 79% of the total cell line. We used the FACSAria I cell sorter to sort the three CD338 cell subsets to explore and compare their stem-like and tumorigenic properties. The figure shows an example of cell sorting of the three subsets based on the image produced by the LSR II analyser. Left panel: surface expression of CD338 in the HCC1937 cell line before cell sorting. Right panels: surface expression analysis of CD338 in the three sorted cell substs. (b) Relative mRNA expression levels of *ABCG2* in CD338^high^, CD338^low^ and CD338^neg^ sorted cell populations as assessed by q-RT-PCR. Levels expressed relative to the housekeeping HPRT1 gene transcript were normalized with respect to the unsorted parental cells ± SD of triplicates. (PDF 111 KB)

Additional file 4: Figure S3: Cross-contamination between CD338^low^ and CD338^neg^ sorted cell subsets. Cytometry analysis of the expression of CD338 in the CD338^low^ and CD338^neg^ sorted cell subsets. The rectangle shows the overlap between the two cell populations. (PDF 49 KB)

Additional file 5: Figure S4: Comparison of the mammosphere formation efficiency of CD24^+^ versus CD24^-^ and of CD24^+^/CD338^+^ versus CD24^+^/CD338^-^ sorted cell subpopulations. (a) CD24^+^ and CD24^-^ cells were separated through cell sorting and plated in non-adherent conditions at low density to assess their mammosphere formation efficiency. CD24^+^ cells (upper panels) were able to form mammospheres with a higher efficiency than the CD24^-^ ones (lower panels, mean ± SEM: 5.8 ± 1.0 and 0.5 ± 0.3 respectively; p < 0.005). **(b)** CD24^+^/CD338^+^ and CD24^+^/CD338^-^ cells were separated through double color cell sorting and plated in non-adherent conditions at low density to assess their mammosphere formation efficiency. Among the CD24+ cells, those overexpressing the stem cell marker CD338 (upper panels) were able to form mammospheres with higher efficiency than their CD338- counterparts (lower panels, mean ± SEM: 13.0 ± 1.1 and 1.5 ± 1.2 respectively; p < 0.005). d2, d3 and d6 indicate days after cell sorting and plating in ultra-low adherent conditions. (PDF 452 KB)

Additional file 6: Figure S5: Link between ABCG2 expression and proliferative activity. CD338^high^ and CD338^-/low^ populations have been sorted as described. The same number of cells from the two sorted cell subpopulations was plated and rate of cell growth was evaluated by counting cells every four days for three weeks. (PDF 49 KB)

Additional file 7: Figure S6: Stabilization of the CD338 antigen-antibody interaction by using the protein cross-linker PMPI (a) Effect of cross-linker treatment on cell sorting purity. Analysis of CD338 expression after cell sorting performed without (upper panels) or with (lower panels) the protein cross-linker. **(b)** Effect of cross-linker treatment on colony forming ability of HCC1937 cells. Unsorted cells were either incubated or not with the cross-linker before CD338 staining and their transformation potential was assessed in a colony formation assay. Colonies were counted after three weeks. Number of colonies are indicated for 5 × 10^4^ cells ± SD of triplicates. (PDF 79 KB)

Additional file 8: Figure S7: Surface expression analysis of HCC1937 cell line. (a, b, c) Gating strategy used to define the cell population subsequently analyzed for the expression of surface markers. To exclude dead cells and debris, cells were gated on a two-physical parameters dot plot measuring forward scatter (FSC) *vs* side scatter (SSC) (a). Doublets were excluded by gating cells on FSC-Height *vs* FSC-Area dot plots (b). To exclude dead cells, Sytox Blue negative cells were gated (c). (d) Surface marker expression analysis on cells gated as described. The expression of each antigen is represented on a frequency distribution histogram (count vs FITC or PE signal). The vertical marker on each histogram used to detect the antibody-positive cells was established using the appropriate negative controls. (PDF 289 KB)

Additional file 9: Figure S8: Cell sorting purity of the different CD24 cell subpopulations. Cells were stained with an anti-CD24 antibody. CD24^+^ and CD24^-^ cell populations were sorted out as described. The left panel shows the cell sorting purity of CD24^+^ subset, the right panel shows the cell sorting purity of CD24^-^. (PDF 50 KB)

## Abbreviations

ABCG2: ATP-Binding Cassette G2
CD: Cluster of differentiation
DAPI: 4′,6-diamidino-2-phenylindole
DMEM: Dulbecco’s modified eagle medium
EDTA: Ethylenediaminetetraacetic acid
EGF: Epidermal Growth Factor
EMT: Epithelial-mesenchymal transition
EpCAM: Epithelial Cell Adhesion Molecule
ER: Estrogen Receptor
ESA: Epithelial specific Antigen
FACS: Fluorescence-Activated Cell Sorting
FBS: Fetal bovine serum
FITC: Fluorescein isothiocyanate
FSC: Forward Scatter
FSC-A: Forward scatter-Area
FSC-H: Forward scatter-Height
H-CAM: Homing-associated Cell Adhesion Molecule
HER2: Human Epidermal Growth Factor Receptor 2
HLA: Human Leukocyte Antigen
IMDM: Iscove’s Modified Dulbecco’s Medium
MFI: Mean Fluorescence Intensity
MoAb: Monoclonal Antibody
NOD/SCID: Nonobese Diabetic/Severe Combined Imunodeficincy
PE: Phycoerythrin
PE-Cy7: Phycoerythrin-Cyanin 7
PMPI: p-Maleimidophenyl isocyanate
RPMI: Roswell Park Memorial Institute-1640 medium
SD: Standard Deviation
SEM: Standard Error of the Mean
SSC: Side Scatter
TIC: Tumor-initiating cell.
