# Supplementary material for: ABCG2, a novel antigen to sort luminal progenitors of BRCA1- breast cancer cells
Source: Mol Cancer. 2014 Sep 12;13:213. doi: 10.1186/1476-4598-13-213 (PMC4176869; doi:10.1186/1476-4598-13-213)
Supplement: Supplementary file 1 — Additional file 1: Table S1: Four-color flow cytometry panel for the expression analysis of surface markers in BT20, HS578T and HCC1937 breast cancer cell lines. (PDF 4 KB) [file 12943_2014_1419_MOESM1_ESM.pdf]

Additional file 1: Table S1

Tube	FITC	PE	PE/Cy7	AlexaFluor647
**1**	CD49f	CD338	CD44	CD24
**2**	CD90	CD133	CD44	CD24
**3**	CD47	CD200	CD44	CD24
**4**	CD227	CD10	CD44	CD24
**5**	CD324	CD29	CD44	CD24
**6**	CD66c	CD61	CD44	CD24
**7**	CD49b	CD184	CD44	CD24
**8**	CD164	CD340	CD44	CD24
**9**	CD9	CD54	CD44	CD24
**10**	CD26	CD55	CD44	CD24
**11**	CD81	CD151	CD44	CD24
**12**	CD66b	CD59	CD44	CD24
**13**	CD165	CD166	CD44	CD24
**14**	CD326	CD105	CD44	CD24
