# Supplementary figures and images for: ABCG2, a novel antigen to sort luminal progenitors of BRCA1- breast cancer cells
Source: Mol Cancer. 2014 Sep 12;13:213. doi: 10.1186/1476-4598-13-213 (PMC4176869; doi:10.1186/1476-4598-13-213)

## Additional file 2: Figure S1

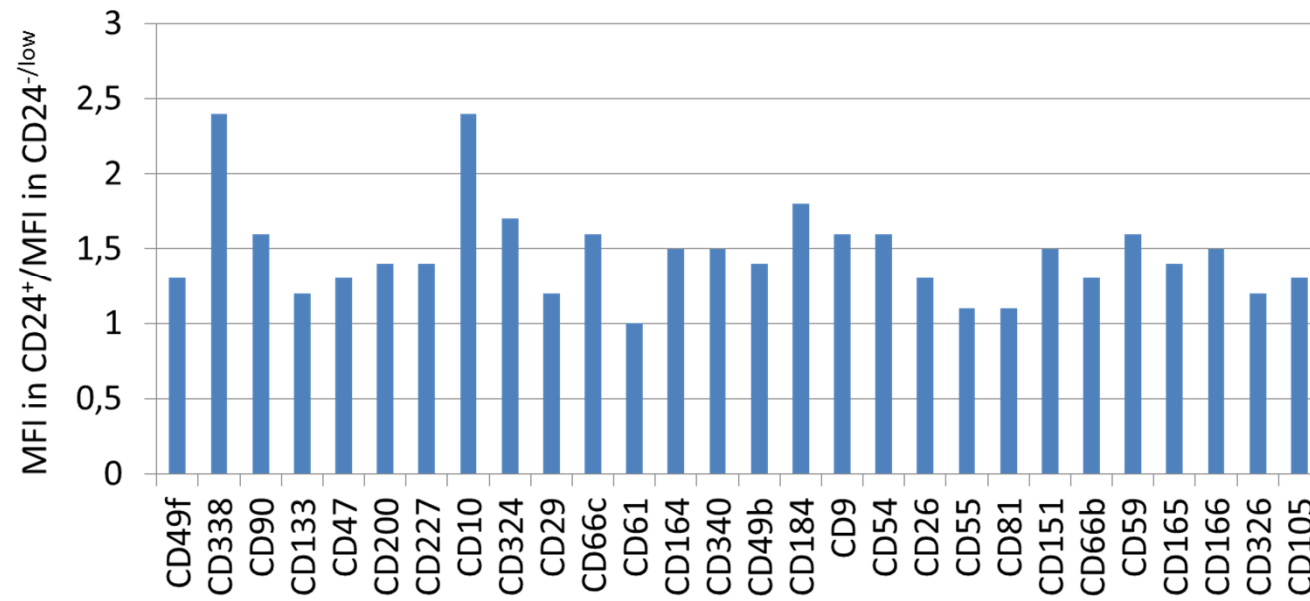

Supplement: Supplementary file 2 — Additional file 2: Figure S1: Expression of surface antigens in the CD24+ and CD24-/low cell subpopulations of the HCC1937 cell line. The surface expression of the breast cancer cell lines was evaluated using a four-color flow-cytometry panel. In particular, we stained cells with monoclonal antibodies against the two classical breast cancer stem cell markers, CD44 and CD24, combined with pairs of antibodies against molecules explored as potential novel TICs markers. Hence, we analyzed and compared the expression of 28 surface markers in the CD44+/CD24-/low and CD44+/CD24+ cell subpopulations of each cell line. This figure shows the ratio of the mean fluorescence intensity (MFI) of each antigen evaluated in the two cell subpopulations, CD24+ and CD24-, present in the HCC1937 cell line. Most CDs displayed a ratio near to 1, which indicated an equal or very similar expression in the two cell subpopulations, whereas CD338/ABCG2 and CD10/CALLA, which are two known stem cell markers, displayed a ratio greater than 2, which indicated that they are expressed at a higher level in the CD24+ than in CD24- cell subpopulation. (PDF 143 KB) [file 12943_2014_1419_MOESM2_ESM.pdf]

## Additional file 3: Figure S2

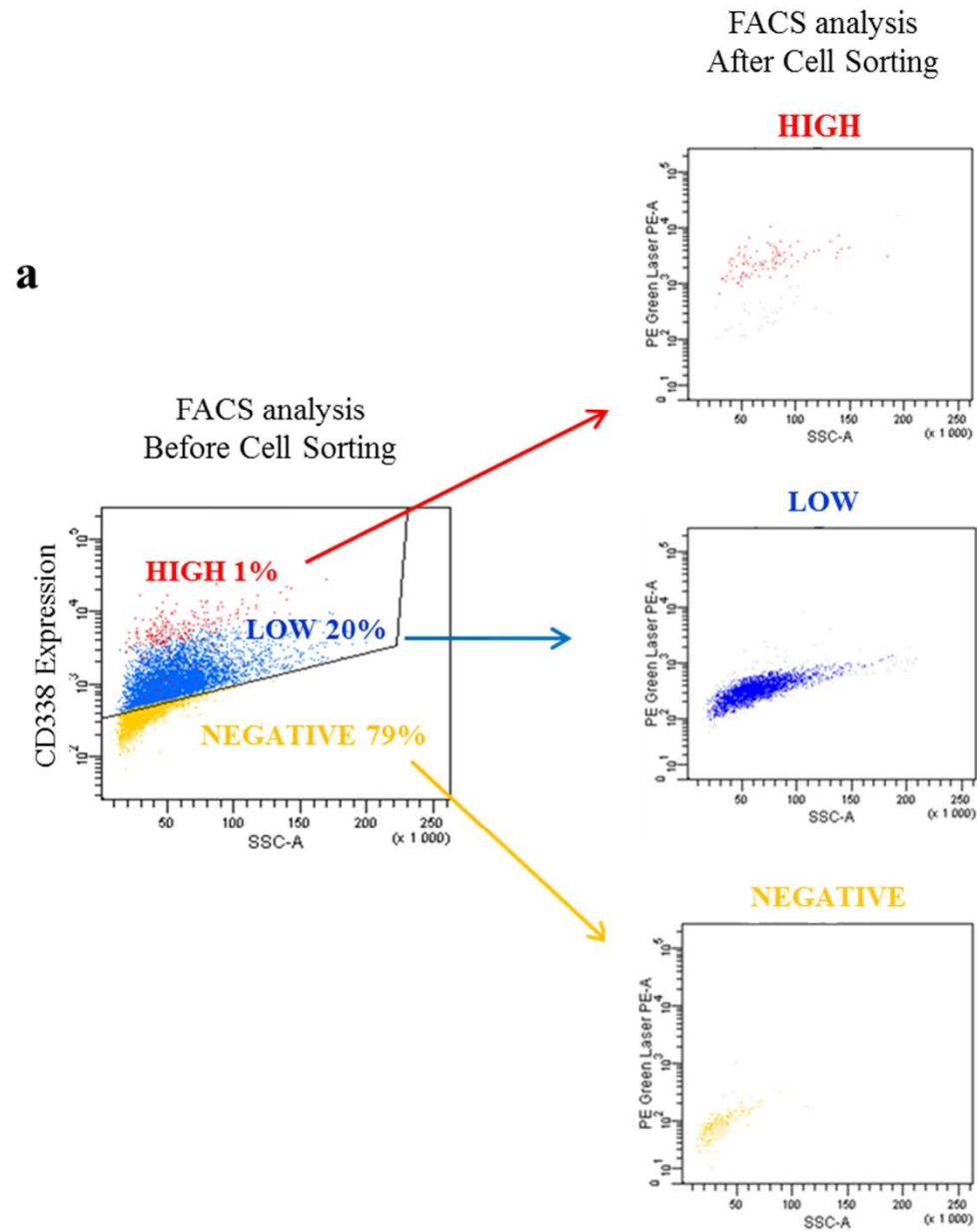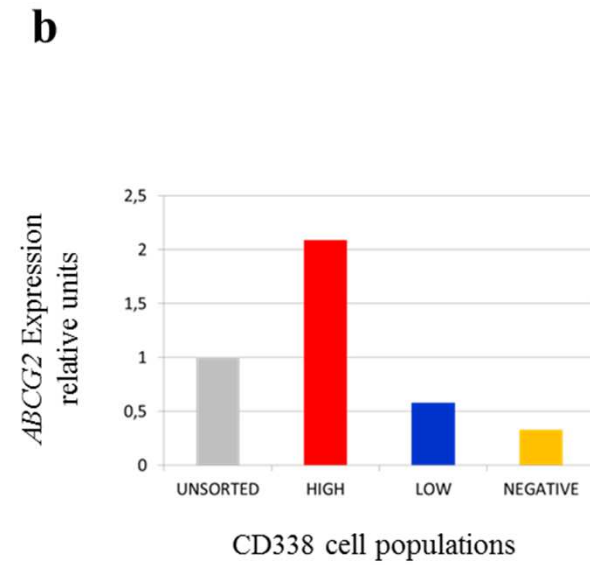

Supplement: Supplementary file 3 — Additional file 3: Figure S2: Expression of CD338 in the HCC1937 cell line and cell sorting of three cell subsets. (a) Using the LSR II cytometer, we identified three distinct CD338 subpopulations: 1) CD338+/high (red events) expressing CD338 at high level and consisting just in 1% of the total cell line; 2) CD338neg (yellow events) not expressing CD338 and constituting about 20% of the total cell line; and 3) CD338+/low (blue events) expressing CD338 at an intermediate level and constituting about 79% of the total cell line. We used the FACSAria I cell sorter to sort the three CD338 cell subsets to explore and compare their stem-like and tumorigenic properties. The figure shows an example of cell sorting of the three subsets based on the image produced by the LSR II analyser. Left panel: surface expression of CD338 in the HCC1937 cell line before cell sorting. Right panels: surface expression analysis of CD338 in the three sorted cell substs. (b) Relative mRNA expression levels of ABCG2 in CD338high, CD338low and CD338neg sorted cell populations as assessed by q-RT-PCR. Levels expressed relative to the housekeeping HPRT1 gene transcript were normalized with respect to the unsorted parental cells ± SD of triplicates. (PDF 111 KB) [file 12943_2014_1419_MOESM3_ESM.pdf]

Additional file 4: Figure S3

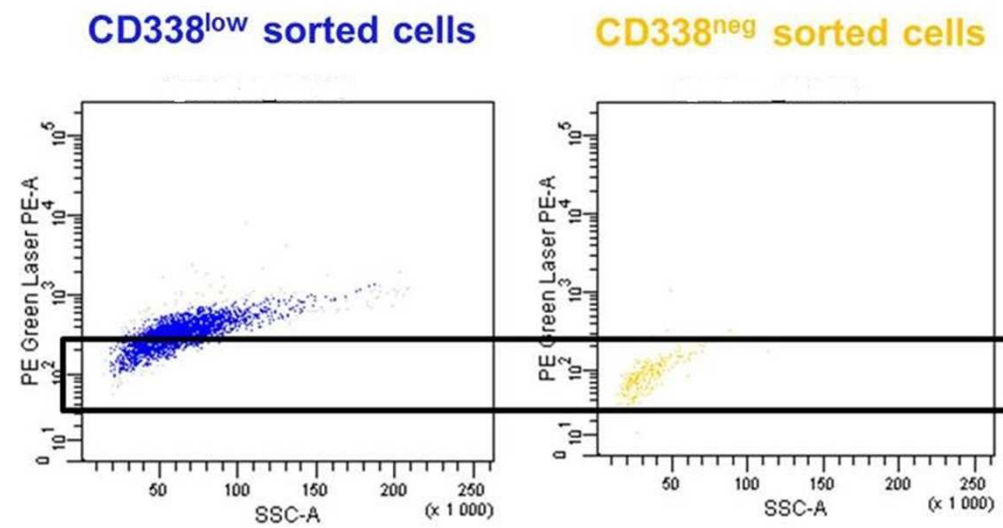

Supplement: Supplementary file 4 — Additional file 4: Figure S3: Cross-contamination between CD338low and CD338neg sorted cell subsets. Cytometry analysis of the expression of CD338 in the CD338low and CD338neg sorted cell subsets. The rectangle shows the overlap between the two cell populations. (PDF 49 KB) [file 12943_2014_1419_MOESM4_ESM.pdf]

## Additional file 5: Figure S4

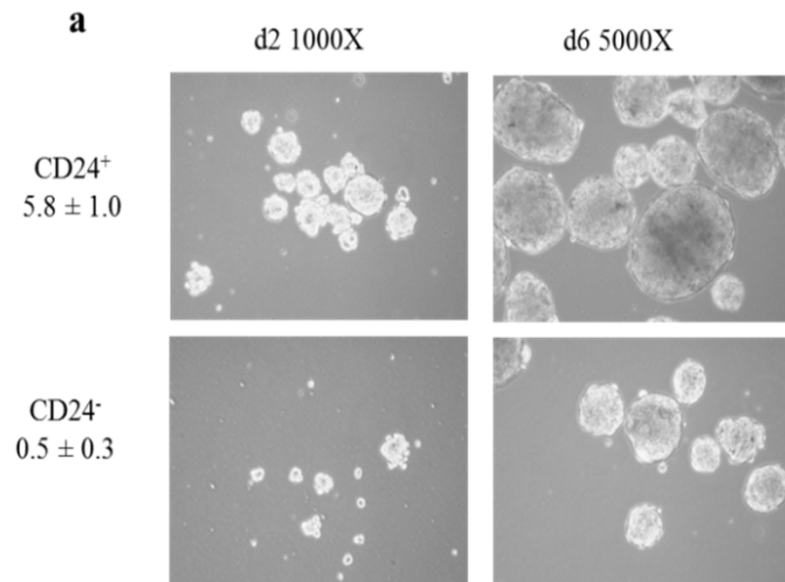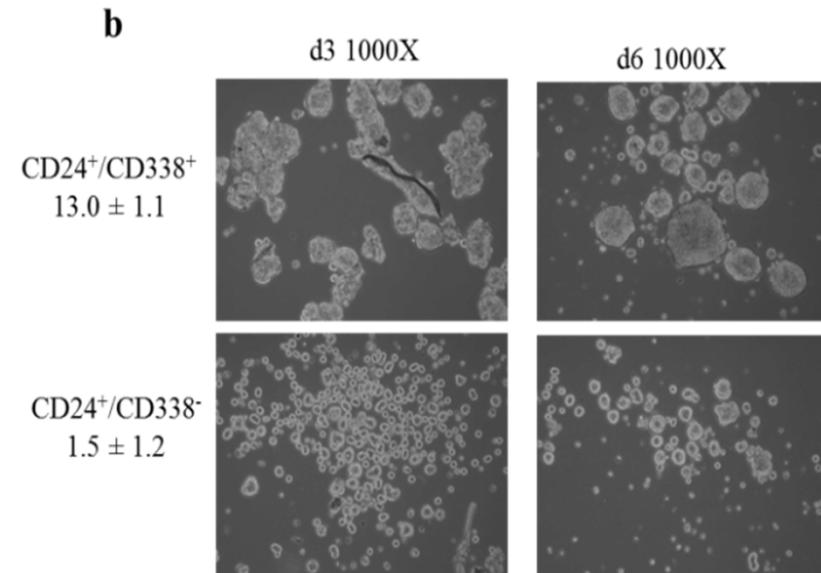

Supplement: Supplementary file 5 — Additional file 5: Figure S4: Comparison of the mammosphere formation efficiency of CD24+ versus CD24- and of CD24+/CD338+ versus CD24+/CD338- sorted cell subpopulations. (a) CD24+ and CD24- cells were separated through cell sorting and plated in non-adherent conditions at low density to assess their mammosphere formation efficiency. CD24+ cells (upper panels) were able to form mammospheres with a higher efficiency than the CD24- ones (lower panels, mean ± SEM: 5.8 ± 1.0 and 0.5 ± 0.3 respectively; p < 0.005). (b) CD24+/CD338+ and CD24+/CD338- cells were separated through double color cell sorting and plated in non-adherent conditions at low density to assess their mammosphere formation efficiency. Among the CD24+ cells, those overexpressing the stem cell marker CD338 (upper panels) were able to form mammospheres with higher efficiency than their CD338- counterparts (lower panels, mean ± SEM: 13.0 ± 1.1 and 1.5 ± 1.2 respectively; p < 0.005). d2, d3 and d6 indicate days after cell sorting and plating in ultra-low adherent conditions. (PDF 452 KB) [file 12943_2014_1419_MOESM5_ESM.pdf]

Additional file 6: Figure S5

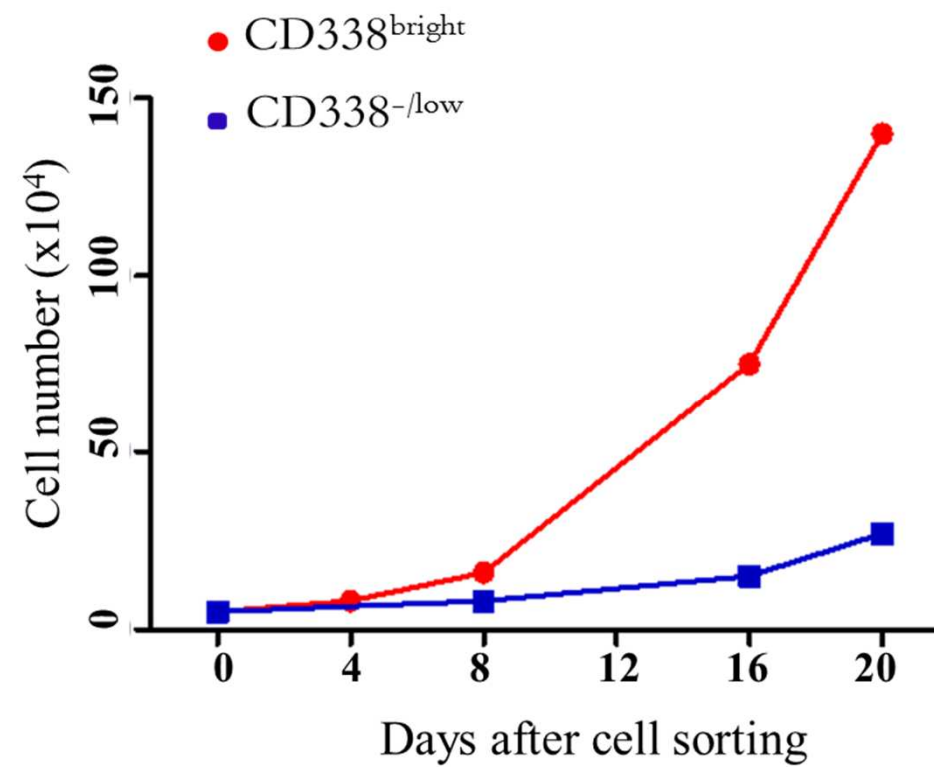

Supplement: Supplementary file 6 — Additional file 6: Figure S5: Link between ABCG2 expression and proliferative activity. CD338high and CD338-/low populations have been sorted as described. The same number of cells from the two sorted cell subpopulations was plated and rate of cell growth was evaluated by counting cells every four days for three weeks. (PDF 49 KB) [file 12943_2014_1419_MOESM6_ESM.pdf]

## Additional file 7: Figure S6

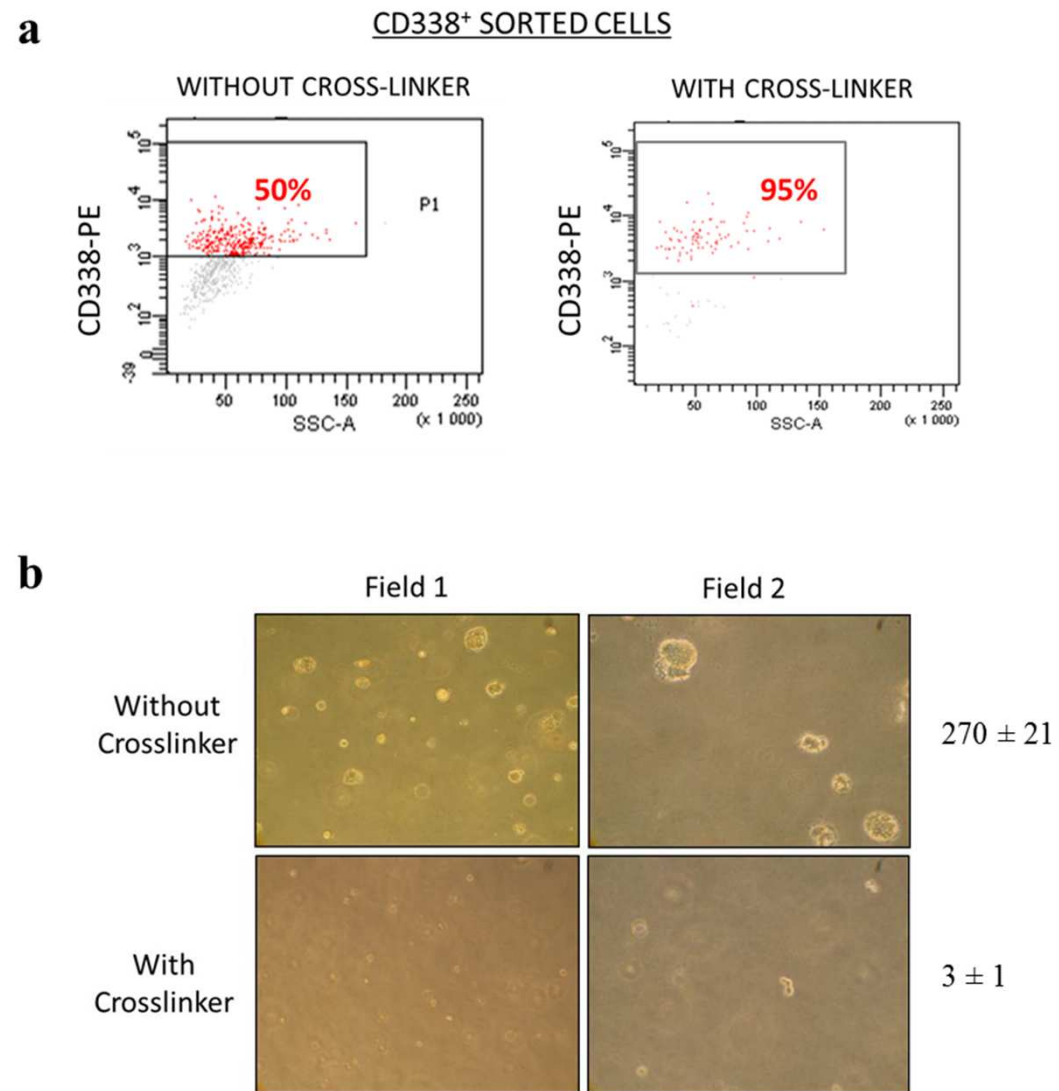

Supplement: Supplementary file 7 — Additional file 7: Figure S6: Stabilization of the CD338 antigen-antibody interaction by using the protein cross-linker PMPI (a) Effect of cross-linker treatment on cell sorting purity. Analysis of CD338 expression after cell sorting performed without (upper panels) or with (lower panels) the protein cross-linker. (b) Effect of cross-linker treatment on colony forming ability of HCC1937 cells. Unsorted cells were either incubated or not with the cross-linker before CD338 staining and their transformation potential was assessed in a colony formation assay. Colonies were counted after three weeks. Number of colonies are indicated for 5 × 10^4^ cells ± SD of triplicates. (PDF 79 KB) [file 12943_2014_1419_MOESM7_ESM.pdf]

# Additional file 8: Figure S7

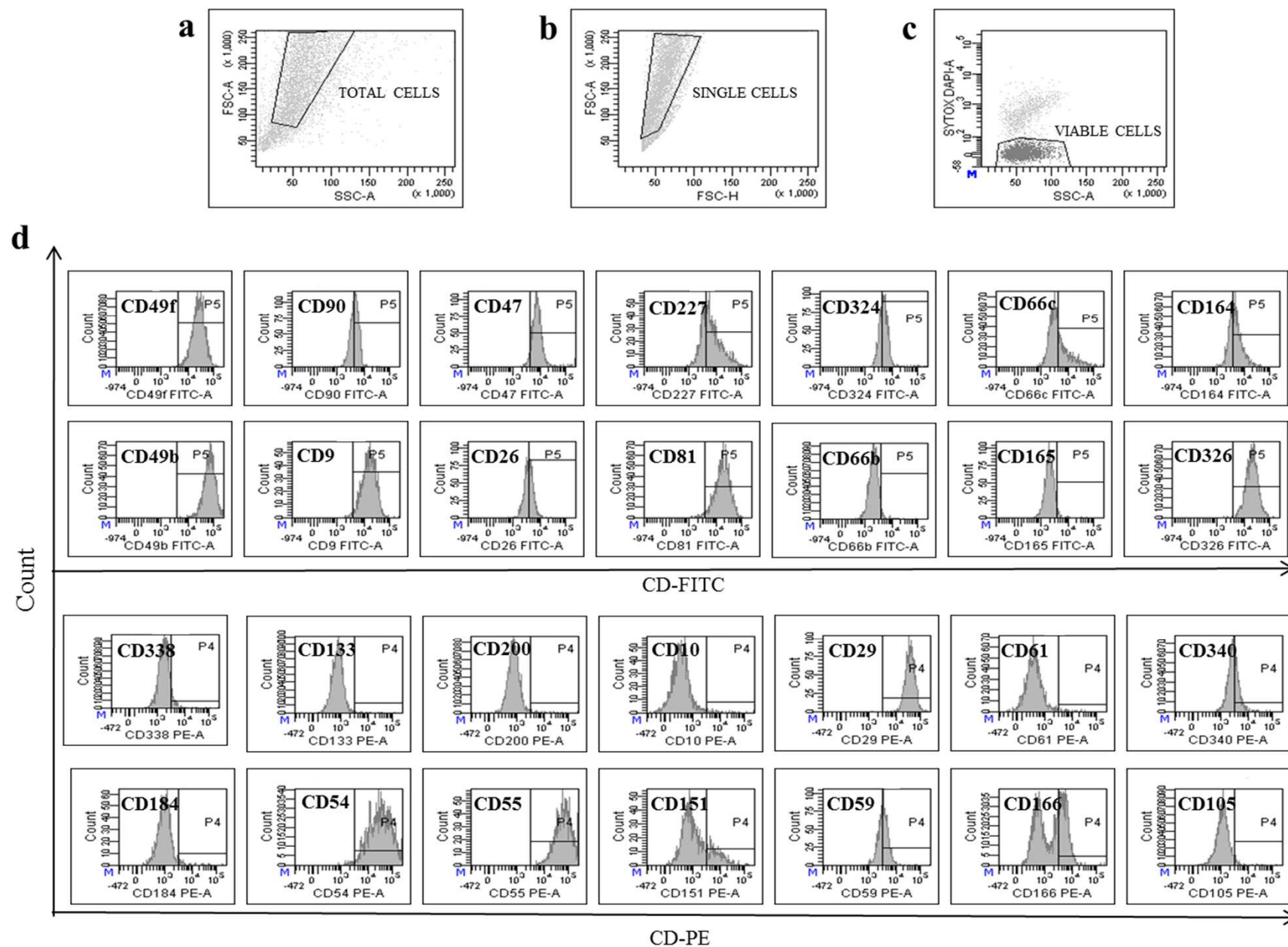

Supplement: Supplementary file 8 — Additional file 8: Figure S7: Surface expression analysis of HCC1937 cell line. (a, b, c) Gating strategy used to define the cell population subsequently analyzed for the expression of surface markers. To exclude dead cells and debris, cells were gated on a two-physical parameters dot plot measuring forward scatter (FSC) vs side scatter (SSC) (a). Doublets were excluded by gating cells on FSC-Height vs FSC-Area dot plots (b). To exclude dead cells, Sytox Blue negative cells were gated (c). (d) Surface marker expression analysis on cells gated as described. The expression of each antigen is represented on a frequency distribution histogram (count vs FITC or PE signal). The vertical marker on each histogram used to detect the antibody-positive cells was established using the appropriate negative controls. (PDF 289 KB) [file 12943_2014_1419_MOESM8_ESM.pdf]

## Additional file 9: Figure S8

CD24<sup>+</sup> sorted cells

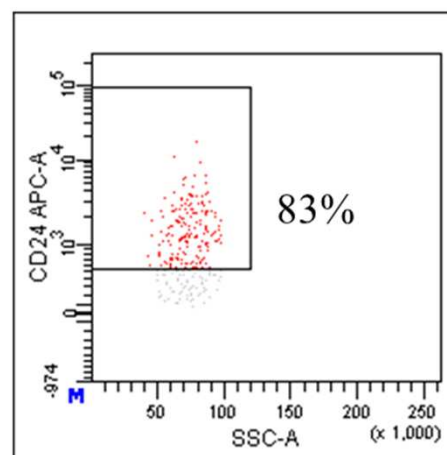

CD24<sup>-</sup> sorted cells

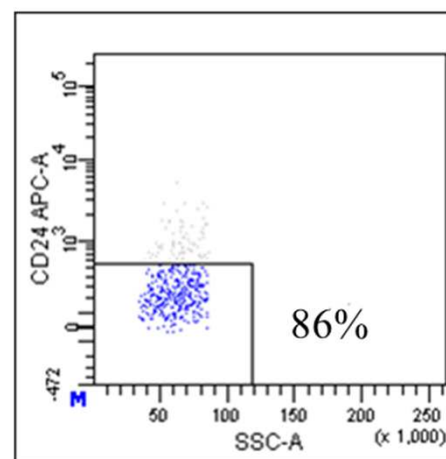

Supplement: Supplementary file 9 — Additional file 9: Figure S8: Cell sorting purity of the different CD24 cell subpopulations. Cells were stained with an anti-CD24 antibody. CD24+ and CD24- cell populations were sorted out as described. The left panel shows the cell sorting purity of CD24+ subset, the right panel shows the cell sorting purity of CD24-. (PDF 50 KB) [file 12943_2014_1419_MOESM9_ESM.pdf]
